# Within-person coupling of estradiol, testosterone, and cortisol in women athletes

**DOI:** 10.7717/peerj.8402

**Published:** 2020-01-24

**Authors:** David A. Edwards, Bulent Turan

**Affiliations:** 1 Department of Psychology, Emory University, Atlanta, GA, United States of America; 2 Department of Psychology, University of Alabama at Birmingham, Birmingham, AL, United States of America

**Keywords:** Estradiol, Testosterone, Cortisol, Hormone coupling, Athletic competition, Stress

## Abstract

**Purpose:**

In variety of settings cortisol and testosterone are positively “coupled.” That is, within-person fluctuations of cortisol and testosterone levels occur in parallel—increases and decreases in one hormone are associated with corresponding increases and decreases in the other. The present report explored hormone coupling in women athletes in two studies selected because they included measurements of salivary levels of cortisol, testosterone, and estradiol—a hormone that has been only infrequently studied in the context of competitive athletics.

**Methods:**

Consenting members of Emory University’s varsity volleyball and soccer teams gave saliva samples on multiple occasions in the run-up to and over the course of two different intercollegiate contests.

**Results:**

Volleyball and soccer players showed remarkably similar hormone-specific patterns of increase in relationship to the different stages of competition—before warm-up, after warm-up, and after competition. For both the volleyball and soccer team, Hierarchical Linear Model (HLM) analyses showed estradiol as being significantly coupled with testosterone which was also coupled with cortisol.

**Conclusions:**

This is, apparently, the first report of significant within-person coupling between estradiol and testosterone in the context of competitive athletic stress. These two hormones may be coupled in a wide variety of circumstances not limited to ones involving sport competition, and results reported here should encourage exploration of the extent to which coordinated fluctuations in estradiol, testosterone, and cortisol levels are present in other, more neutral settings and the ways in which the coordination of these fluctuating hormone levels may benefit human performance.

## Introduction

Cortisol is produced in the adrenal cortex which also secretes the androgenic steroid testosterone and androgen precursors. These secretions are regulated by the hypothalamic-pituitary-adrenal (HPA) axis. Testosterone is additionally produced and secreted by the testes and ovaries which also secrete estradiol and other estrogens. These processes are controlled by the hypothalamic-pituitary-gonadal (HPG) axis. Additional amounts of estradiol may be contributed by the peripheral aromatization of testosterone. While cortisol and testosterone can inhibit the axis of the other (Viau, 2002), in a variety of settings the activation and deactivation of the two systems appear to be positively “coupled.” That is, within-person fluctuations of cortisol and testosterone levels occur in parallel—increases and decreases in one hormone are associated with corresponding increases and decreases in the other. In humans, cortisol/testosterone coupling has been reported for incarcerated adolescent boys (Johnson et al., 2014), a non-institutionalized sample of adolescent boys and girls (Marceau et al., 2015) and a mixed-sex, lifespan sample of individuals ranging in age from 11–88 years (Harden et al., 2016). Cortisol/testosterone coupling may be particularly evident in situations involving social/evaluative stress: Turan et al. (2015) reported positive within-person coupling for adult men and pre/early pubertal boys and girls responding to age-appropriate forms of the Trier Social Stress Test (TSST).

In laboratory studies using the TSST, the magnitude of positive coupling between cortisol and testosterone can be affected by person factors such as dominance, anxiety, and negative affect (Turan et al., 2015). In a small sample of incarcerated adolescent males, psychopathology and callousness were related to cortisol/testosterone coupling, with tighter coupling positively associated with psychopathology and hormone uncoupling associated with callousness (Dismukes et al., 2015).

In a variety of sports, athletic competition is associated with an increase in levels of cortisol and testosterone in men and women (Casto & Edwards, 2016a for a general review; Slimani et al., 2017 for a review focused on soccer). At least for women, individual differences in cortisol and testosterone reactivity in sport settings are positively correlated—before to after-competition increases in one hormone are positively correlated with increases in the other (Edwards & Casto, 2013). That levels of each hormone change in connection with competition make athletic contests an appealing setting for the study of hormone coupling.

In one report (Stanton & Schultheiss, 2007) salivary levels of estradiol and testosterone measured in women prior to engaging in a non-athletic contest were positively correlated with each other and estradiol (but not testosterone) was positively related to implicit power motivation. Levels of estradiol in women soccer players rise in anticipation of an upcoming match and continue to increase during the warm-up period prior to it (Casto & Edwards, 2016b). Correlations between testosterone and estradiol assayed from facial and axillary perspiration in men and women are high (Elliot, Muir & De Catanzaro, 2017; Muir et al., 2008). In naturally cycling women, serum testosterone and estradiol levels peak together at mid-cycle (Rothman et al., 2011) and strenuous aerobic running increases plasma levels of testosterone and estradiol (Kuoppasalmi et al., 1976). Taken together, these results raise the possibility that within-individual fluctuations in these two hormones are positively coupled. The present study documents the coupling of testosterone with estradiol and cortisol in connection with competitive athletics, and provides new information about intra-individual consistency in hormone (estradiol, testosterone, cortisol) reactivity from one athletic contest to another.

## Materials & Methods

For more than a decade, one of us (DE) has worked with women athletes on studies of the hormonal correlates of intercollegiate competition. For the present report we retrospectively examined data for two of these, selected because participants in each contributed a relatively large number of saliva samples and assay results included values for estradiol as well as for testosterone and cortisol. Portions of the data sets for the two studies were used in the preparation of two articles (Casto & Edwards, 2016b; Edwards & Kurlander, 2010) about hormones and athletic competition in women.

Participants were the 14 active and consenting members of the Emory University (Atlanta, GA) women’s 2008 varsity volleyball team and the 25 consenting members of the 2013 varsity women’s soccer team, giving a total sample size of 39 women. Four members of the volleyball team and 16 members of the soccer team were using oral contraceptives. Women ranged in age from 18–22 years. The data do not include values for two additional consenting participants (both volleyball players), one who suffered a season-ending injury prior to the start of the study and another who suffered an injury during competition forcing her to leave the match. Each study was approved by Emory’s Institutional Review Board (volleyball: IRB no. 00012665; soccer: IRB no. 00068541). Details about the consent process as well as queries about participant use of hormone contraceptives can be found elsewhere (Casto & Edwards, 2016b; Edwards & Kurlander, 2010).

### Saliva samples and hormone assays

For the volleyball team we collected saliva samples before and after each of two practice sessions beginning at 3:15 PM and ending around 5:40 PM on two consecutive days in mid-October. In addition, we collected saliva samples in connection with two matches played one day apart on October 31st and November 1st. An initial game-day sample was collected as athletes dressed for their match 30–40 min before the start of an hour-long warm-up. A second sample was obtained mid-warm-up and a third sample was obtained immediately after the end of each match. This makes a total of 10 saliva samples for each participant with the exception of instances where players either did not give a sufficient volume of saliva for assay or there was not enough product remaining for estradiol assay after the completion of assays for testosterone and cortisol. The first match (a 3–0 victory) began at 7:10 PM and was against a Division III opponent, at the time ranked first in the country. The match was completed at 8:30 PM. The second match (a 0–3 loss against a Division II opponent) began at noon and was completed at 1:10 PM. Emory would go on to finish the season as National Collegiate Athletic Association, Division III champion.

**Figure 1:**
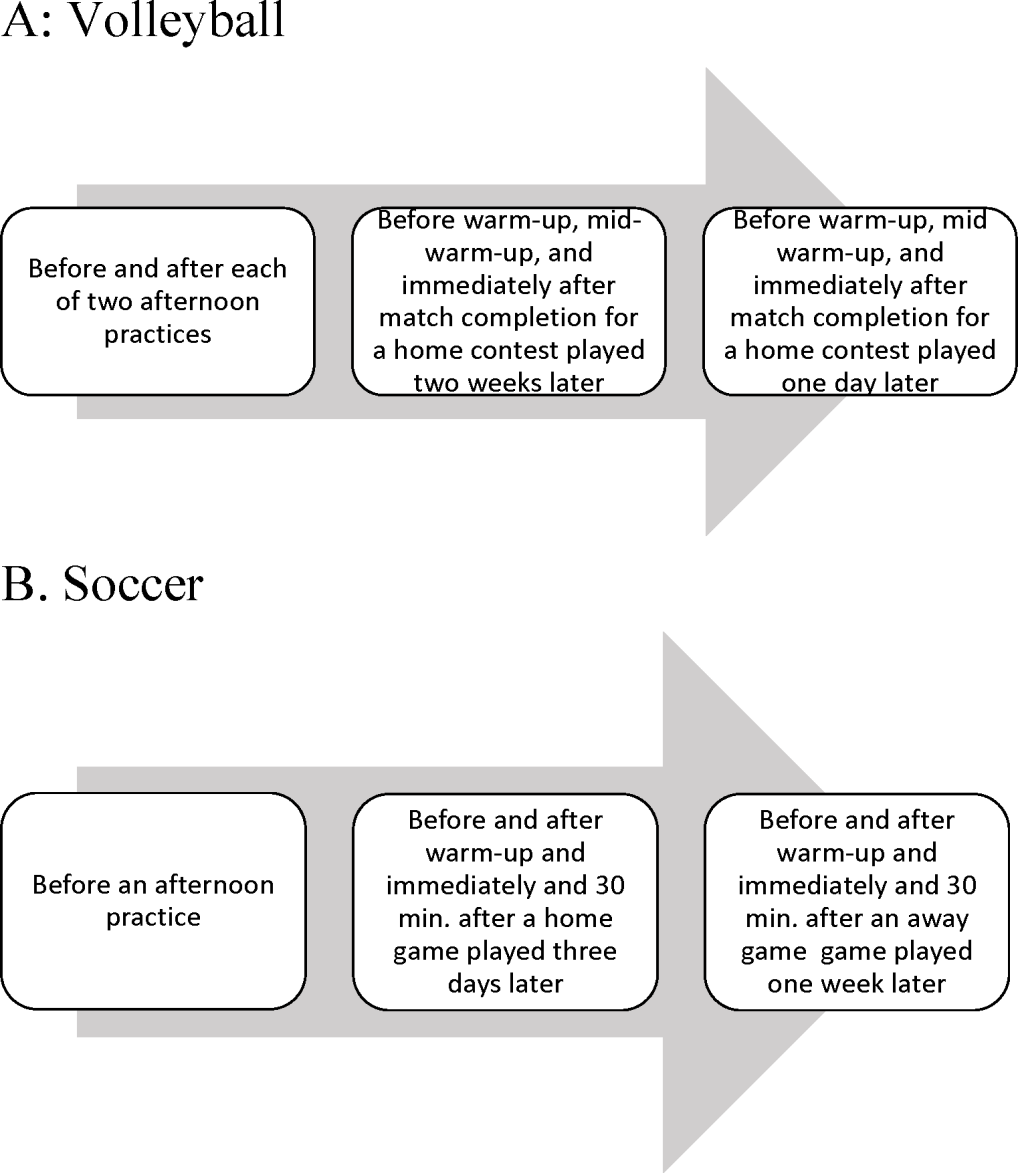
Timeline for collection of saliva samples. (A) Volleyball team. (B) Soccer team.

For the soccer team, five to nine samples were obtained from each participant. All participants gave one neutral-day baseline sample at the time of consent before an afternoon practice session three days before the first match. Then, participants gave four samples in association with each of two intra-conference NCAA soccer competitions one week apart in October. All samples were collected between 2 and 4 PM. The first of the two matches (a 0–1 loss) was played at the participants’ home field; the second game (a 2–0) victory was played away from home. For each match, participants gave a saliva sample 10–15 min before the start of an hour-long warm-up, another sample immediately after warm-up (a few minutes before the start of the match), a third sample immediately after match completion, and a final sample 30 min later. Not all the participants were part of the travelling squad; for these individuals we only had five saliva samples for each individual. Figure 1 shows the timeline for collection of saliva samples for volleyball and soccer players. Hormone values for soccer and volleyball teams are available as supplemental files (S1 and S2, respectively).

Participants gave samples by drool exactly according to protocols published elsewhere (Casto & Edwards, 2016b; Edwards & Kurlander, 2010). Samples were placed on ice, frozen within the hour, and stored (volleyball: −26 °C; soccer: −80 °C) until assay.

All assays were conducted by the Biomarkers Core Laboratory of the Yerkes Primate Center (Atlanta, GA). For the volleyball study, cortisol and testosterone were assayed using kits from Diagnostics Systems Laboratories (Webster, TX); assays for estradiol were performed using kits produced by American Laboratory Products Company (Windham, NH). For the soccer study, samples were assayed on a single thaw for cortisol, testosterone, and estradiol using kits from Salimetrics (State College, PA). For additional details (Casto & Edwards, 2016b; Edwards & Kurlander, 2010).

### Statistical analyses

Because of differences in the total number of samples collected, the times of collection, the assay kits used for hormone analysis, and differences in the inherent character of volleyball and soccer competitions, the data sets for the volleyball and soccer teams were analyzed separately. Participants gave multiple saliva samples. Changes in hormone levels from neutral-day baseline through different stages of athletic competition were analyzed using the SPSS statistical package for repeated measures ANOVA that includes paired comparisons (without adjustment for confidence intervals) of competition-stage levels with baseline. Effect sizes for these comparisons are represented by Cohen’s *d*. Product-moment correlations were used to analyze for consistency of hormone reactivity from one competition to another.

For each study, within-person coupling between each possible pair of hormones (cortisol and testosterone, cortisol and estradiol, testosterone and estradiol) was examined using hierarchical linear modeling (HLM) version 6.08 (Raudenbush, Bryk & Congdon, 2004). Three-level HLM analyses were conducted (hormone samples clustered within days, days clustered within participants). Oral contraceptive use was controlled in each model. In all analyses, Level 1 (within-participant level) variables were group-centered (the variable mean for each participant across all hormone samples was subtracted from that participant’s hormone level at each time point) and Level 3 variables were grand centered (the mean variable score for all participants was subtracted from each participant’s score). All parameters at Level 2 and Level 3 were specified as random to allow for variability between participants. Then, as a check on the robustness of the findings we repeated all analyses for the subset of athletes who played in both games (Volleyball, *N* = 9; Soccer, *N* = 14). For every participant, within-person product-moment correlations were also calculated for every possible hormone pair to provide easy-to-visualize measures of within-individual hormone coupling that could be compared across participants.

## Results

### Descriptive statistics

**Figure 2:**
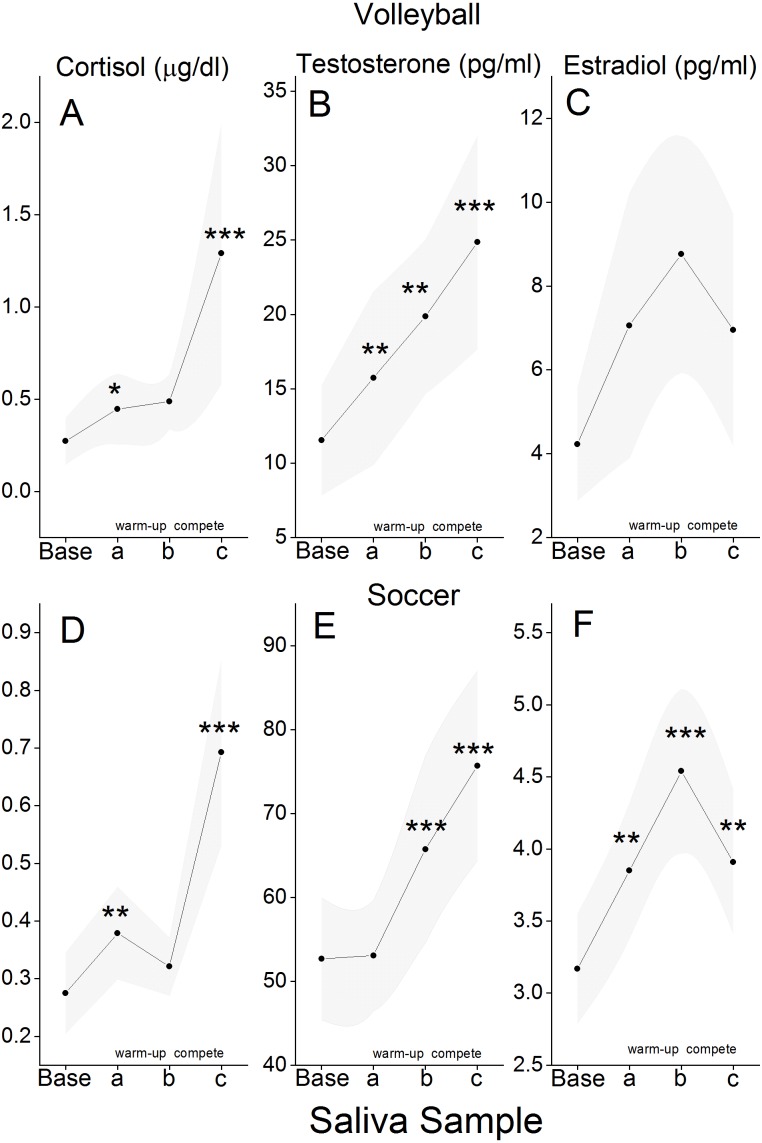
Mean hormone values for different stages of competition. Saliva samples were obtained on a neutral-day (Base) and at three different times on the day of competition. Sample a was obtained before warm-up; sample b was obtained either mid-warm-up (Volleyball) or immediately after the end of warm-up (Soccer); sample c was obtained immediately after the end of competition. Group means (Cortisol: (A) and (D); testosterone: (B) and (E); estradiol: (C) and (F)) are for those individuals who actually played in the competition (Volleyball, *N* = 9; Soccer, *N* = 17). Ninety-five percent confidence intervals are shown as shaded bands. Symbols (^∗^*p* ≤ .05; ^∗∗^*p* ≤ .02; ^∗∗∗^*p* ≤ .01) represent instances where the group mean is significantly different from the one for the preceding sample. Cohen’s d values for statistically significant differences ranged from .64 to 2.4. Team differences with respect to mean hormone levels are likely attributable to the fact that assays for the volleyball and soccer teams were performed with kits from two different manufacturers.

For the volleyball and soccer teams, there were four points of correspondence with respect to hormone values. First was neutral-day baseline –hormone values determined by analysis of samples obtained in the afternoon before practice (for the volleyball players we averaged the values for saliva samples obtained before the start of each of two practice sessions; the soccer players had only one before-practice sample). Then, there were hormone values for three corresponding competition-day samples. Participants gave their first competition-day sample before warm-up (Sample A), gave a second sample at either mid-warm-up (volleyball), or shortly after warm-up (soccer) (Sample B), and a third sample immediately after the end of the competition (Sample C). For purposes of between-team comparisons, we selected the first of the two contests played by each team so that for both teams, competition-day samples would be for a contest played at home. Figure 2 shows mean cortisol, testosterone, and estradiol values for individuals who actually played in the competition (volleyball: *N* = 9; soccer: *N* = 17). Salivary levels of each hormone change in hormone-specific patterns in relationship to the stage of competition, and the patterns are remarkably similar for the two sports. Cortisol levels increased from neutral-day baseline to the day of competition (before warm-up), remained essentially unchanged during the warm-up period, and increased dramatically during the period of actual competition. Testosterone levels increased from neutral-day to the day of competition for volleyball players but remained at baseline levels for soccer players. But, for both volleyball and soccer players, testosterone increased during warm-up and continued to increase during competition. Estradiol levels increased from neutral-day to competition day and increased further during warm-up, but decreased during the period of actual competition.

### Individual differences in hormone reactivity to competition

As a measure of reactivity to competition, for every individual who played in both competitions, we took the maximum (peak) values for cortisol, estradiol, and testosterone without reference to stage of competition (before warm-up; after warm-up; immediately after competition) and expressed these values as a percent of neutral-day baseline value. Individual differences in cortisol, testosterone, and estradiol reactivity were generally conserved across the two competitions. This is most clearly evident for the soccer players, where reactivity scores for the two competitions were highly correlated (cortisol, r (12) = .71, p = .004; testosterone, r (12) = .86, *p* < .001 ; and estradiol, r (11) = .61, *p* = .03). Results were in the same direction for the volleyball players but, perhaps owing to smaller sample size, only the Match 1/Match 2 reactivity correlation for testosterone was statistically significant (r(7) = .86, p = .003). Are individual differences in reactivity for one hormone related to individual differences in reactivity to another? To answer this question, we considered each of the two athletic contests separately for the volleyball players and soccer players, including in our analysis only individuals who actually played in the contest being considered. The correlation between cortisol and testosterone reactivity for the soccer players was statistically significant (Game 1: r(15) = .69, p = .002; Game 2: r(13) = .52, *p* = .046). With r-values ranging between .24 and −.09, none of the reactivity comparisons between estradiol and testosterone, and cortisol and estradiol were statistically significant for either the volleyball or soccer players.

### Multi-level (HLM) analyses: volleyball

Within-person cortisol and testosterone were significantly coupled (coefficient = 12.89, SE = 2.41, *t* = 5.36, *p* < .001), as were within-person estradiol and testosterone (coefficient = 0.65, SE = 0.26, *t* = 2.48, *p* = .03). Within-person cortisol and estradiol were not significantly coupled (coefficient = 0.03, SE = 0.02, *t* = 1.15, *p* = .16). Oral contraceptive use did not moderate any of the coupling effects. Sensitivity analyses with only those athletes who played in both games yielded similar results: cortisol and testosterone showed a significant positive within-person coupling (coefficient = 11.66, SE = 1.38, *t* = 8.42, *p* < .001) as did estradiol and testosterone (coefficient = 1.07, SE = 0.38, *t* = 2.84, *p* = .03). Within-person cortisol and estradiol were not significantly coupled (coefficient = 0.03, SE = 0.03, *t* = 0.95, *p* = .37).

### Multi-level (HLM) analyses: soccer

Analysis for within-person coupling between cortisol and testosterone was statistically significant (coefficient = 21.89, SE = 4.73, *t* = 4.62, *p* < .001 ) as was the analysis for coupling between estradiol and testosterone (coefficient = 3.41, SE = 1.26, *t* = 2.71, *p* = .01). Within-person coupling between cortisol and estradiol was not statistically significant (coefficient = −0.06, SE = 0.03, *t* =  − 2.00, *p* = .06). Oral contraceptive use did not moderate any of these coupling effects, except for the estradiol and testosterone coupling, which was weaker for those on oral contraceptives (coefficient = −5.44, SE = 2.58, *t* =  − 2.11, *p* = .046). Sensitivity analyses with only those athletes who played in both games yielded similar results. Cortisol and testosterone showed a significant positive within-person coupling average (coefficient = 23.44, SE = 5.35, *t* = 4.38, *p* = .001) as did estradiol and testosterone (coefficient = 4.30, SE = 1.94, *t* = 2.21, *p* = .047). HLM analysis for cortisol and estradiol was not statistically significant (coefficient = −0.08, SE = 0.05, *t* =  − 1.66, *p* = .12).

### Within-person correlations: volleyball and soccer

**Figure 3:**
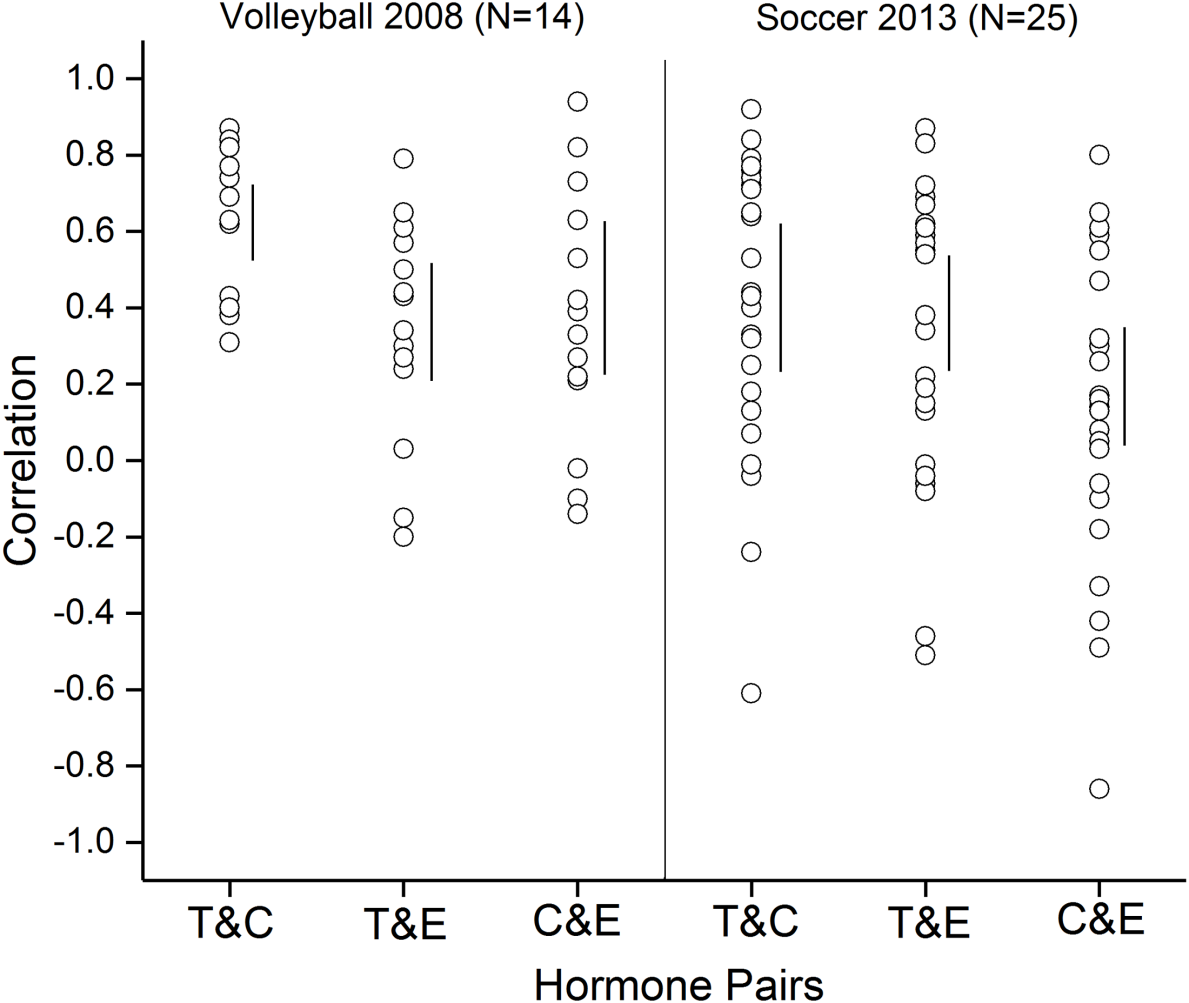
Within-individual correlations between hormone levels. Values represent Pearson correlations between levels of testosterone and cortisol (T&C), testosterone and estradiol (T&E), and cortisol and estradiol (C&E) for each of the participating members of the 2008 volleyball team and the 2013 soccer team. Each data point shows the correlation between levels of two hormones for a single individual. Vertical bars represent 95% confidence intervals for the group means.

Within-person Pearson correlations between cortisol, testosterone, and estradiol across the different time points for saliva sampling are shown in Fig. 3 for each participant. For both volleyball and soccer teams, the majority of individuals show positive correlations between levels of salivary cortisol, testosterone, and estradiol. But, there are large individual differences with respect to the degree to which fluctuating levels of testosterone, estradiol, and cortisol are linked with each other. For any given hormone pair, there are some individuals in which variation in the level of one hormone is almost perfectly linked with variation in another—in some instances the two hormones changed in the same direction, but in others they changed in opposite directions. In still others, changes in levels of hormones appeared to be completely unrelated.

## Discussion

### Competition-related changes in levels of testosterone, cortisol, and estradiol

In accordance with earlier studies of women athletes in a variety of sport settings (Casto & Edwards, 2016a for a review), volleyball and soccer players showed increases in both cortisol and testosterone during the period of actual play. For example, for the volleyball and soccer players whose data are shown in Fig. 2, the vast majority of individuals (76 percent and 89 percent, for volleyball and soccer, respectively) showed an increase in levels of cortisol and testosterone from before warm-up to after competition.

When “competition” is considered in a more temporally extended manner, levels of cortisol, testosterone, and estradiol predictably change in relationship to actual play. Specifically, for both volleyball and soccer players, cortisol and estradiol increased from neutral-day to before warm-up on the day of competition. For volleyball players (but not soccer players) these increases were accompanied by an increase in testosterone. For volleyball and soccer players, testosterone and estradiol increased during warm-up while, on average, levels of cortisol remained stable. For both teams, cortisol and testosterone increased during competition, while estradiol returned to before warm-up levels.

Many of the participants played in each of two intercollegiate competitions held one day (volleyball) to one week (soccer) apart. Whether for cortisol, testosterone, or estradiol, when peak competition-day hormone level is expressed as percent change from neutral-day baseline, individual differences in reactivity to competition appear to be relatively stable from the first competition to the second. Perhaps owing to a larger sample size, this was most in evidence for the soccer players. Writing about cortisol and testosterone, Edwards & Kurlander (2010) suggested that women may have a “signature” change in hormone level that is conserved from one competition to another. The present study makes it clear that this applies to estradiol as well.

Are individual differences in reactivity for one hormone related to individual differences in reactivity to another? The answer to this question is “yes,” but only for the soccer players and only for testosterone/cortisol reactivity. Reactivity scores for estradiol were not significantly correlated with reactivity scores for either testosterone or cortisol. It is important to appreciate that, as we have calculated it, hormone “reactivity” reflects the maximum competition-day hormone change relative to neutral-day baseline without reference to the stage of competition at which this maximum is attained. For this reason, while reactivity scores are useful in comparing individual differences in hormone reactivity to competition, they are different than within-person concurrent fluctuations in levels of cortisol, testosterone, and estradiol. As discussed in the next section, using HLM analyses we found strong evidence for within-person coupling of estradiol and testosterone as well as testosterone and cortisol.

### Within-person coupling of testosterone, cortisol, and estradiol

For both volleyball and soccer teams, HLM analysis showed testosterone as being significantly coupled with cortisol and estradiol. These relationships held when all the players on each team were included in the analysis as well as when the analysis was done with the more limited sample of players who played in both of their team’s two competitions. Although on average cortisol and estradiol were not significantly coupled by HLM analysis, when considered on an individual basis (Fig. 3), fluctuations in cortisol and estradiol levels were highly correlated for some individuals but not for others.

The within-person coupling of testosterone and cortisol in a setting that includes a sport competition is in accordance with earlier reports of testosterone/cortisol coupling in more neutral settings (e.g., Marceau et al., 2015; Harden et al., 2016). Consonant with the coordinated coupling of testosterone and cortisol responses to social evaluative stress (Turan et al., 2015), the results reported here expand the contexts in which coupling of these two hormones has been demonstrated to competitive athletics.

The present study is the first, we believe, to show significant within-person coupling between estradiol and testosterone in the context of a competitive athletic setting, where levels of both hormones are known to vary (e.g., Casto & Edwards, 2016b). This result is consonant with reported correlations between these two hormones in samples of axillary and facial perspiration obtained in laboratory settings (Elliot, Muir & De Catanzaro, 2017; Muir et al., 2008). Consideration of hormone relationships on an individual basis (Fig. 3) provides evidence that at least for some individuals, fluctuations in cortisol and estradiol may also be linked. The sampling of multiple hormones (including estradiol in addition to testosterone and cortisol) in studies of stress and status, and studies of the extent to which coordinated fluctuations in estradiol, testosterone, and cortisol levels are present in other settings are clearly warranted.

For any given pair of hormones there were large individual differences in the strength and direction of their relationship to each other (see Fig. 3). Person variables such as dominance, anxiety, and negative affect, and psychopathological traits can affect the strength of coupling between cortisol and testosterone (e.g., Johnson et al., 2014; Turan et al., 2015). The extent to which these or any other person variables affect estradiol/testosterone coupling remains to be determined. All of the participants in the present study were women. Whether salivary levels of estradiol and testosterone are coupled over the course of an athletic competition in men remains an open question.

### Hormone sources and possible mechanisms for coupling

Cortisol is secreted exclusively by the adrenal cortex, and adrenocortical responses to exercise and other stressors appear to be secondary to an increase in ACTH i.e., activation of the HPA axis (e.g., Lennartsson et al., 2012). With respect to both testosterone and estradiol, the source (ovarian or adrenal), and proximate cause(s) of competition-related increases in salivary levels remain to be determined; possibilities include secretion, decreased metabolic clearance, hemocentration, and sympathetic activation of the ovary (Edwards & Casto, 2013, p.158).

Whether for volleyball or soccer, changing salivary levels of cortisol, testosterone, and estradiol appear to be coordinated in relationship to the period of actual competition and, by the very nature of things, each other. That within-individual fluctuations in cortisol and testosterone are positively related is perhaps because the adrenal cortex is the principal source for elevated levels of testosterone associated with athletic competition. Alternatively, when cortisol and testosterone responses to competition are positively related, it could be because the adrenal glands and gonads are responding similarly to the physical and/or psychological elements of competition. The positive within-individual correlations between fluctuating levels of testosterone and estradiol suggest the ovaries as a common source for these hormones.

Each of these hypotheses is challenged by the fact that competition-related changes in salivary levels of cortisol, testosterone, and estradiol do not occur in perfect parallel. That estradiol levels change in association with competition following their own pattern, at times similar to and at other times different from, the patterns by which testosterone and cortisol change in relationship to competition, argues against a single unifying causal principle (e.g., common source or reduced hepatic degradation) underlying competition-related changes in these hormones. Rather, that testosterone, cortisol, and estradiol levels change differently in the run-up to the day of competition, during warm-up, and moments of actual competition, and that there are large inter-individual differences in the magnitude of the within-individual correspondence between levels cortisol, testosterone, and estradiol, suggests that there are multiple, hormone-specific, psychological and physical factors that, either independently or in association with each other, affect competition-related changes in hormone levels (Casto & Edwards, 2016b, p.23).

Given that group averages for levels of cortisol, testosterone, and estradiol each change in somewhat different hormone-specific patterns in relation to the stages of competition (Fig. 2), it may seem paradoxical that fluctuating levels of cortisol and testosterone, and levels of testosterone and estradiol appear so robustly coupled by HLM analysis. However, it should be noted that HLM analysis tests for within-person coupling of hormone levels, which may be significant even if the trajectories of hormone change for the group differ in the run-up to and over the course of an athletic contest.

### Benefits of hormone coupling

As reviewed in more detail elsewhere (Casto & Edwards, 2016a), a rise in levels of cortisol and testosterone may have physiological and/or psychological effects that benefit performance in athletic competition. For cortisol these include an increase positive affect, an increase in the availability of metabolic fuel, and reduced inflammation in muscle and other tissues. Transient increases in testosterone can increase feelings of dominance, power, or competitiveness. Testosterone and estradiol can have neuroprotective effects (Behl, 2002; Bialek et al., 2004) and it has been suggested (Maya et al., 2016) that increases in testosterone may have immunoprotective effects for participants. Short term increases in estradiol levels can increase vasodilation of coronary arteries and stimulate the production of insulin (Gruber et al., 2002 for review) and could augment feelings of social connectedness among teammates prior to a competition in which their coordinated efforts will be essential for optimum team performance (Casto & Edwards, 2016b). Turan et al. (2015), p. 65) have argued that the coupled relationship between cortisol and testosterone reflects the operation of an evolved system that monitors situations involving one’s social status, “eliciting coordinated psychological, behavioral, and physiological response when a threat to status is detected.”

The coupled relationships between fluctuating levels of testosterone and cortisol on the one hand, and testosterone and estradiol on the other raise the possibility of benefits, the nature of which are presently unknown, accruing from the collective and coordinated elevations of each of these hormones in relationship to the different stages of athletic competition and, perhaps, competition in non-athletic settings as well. A better understanding of these benefits could be exploited to enhance competitive performance. A case in point: levels of testosterone, cortisol, and (presumably) estradiol are subject to influence by selected “motivational” video clips and there is at least one report (Cook & Crewther, 2012) of a video-related increase in testosterone as being closely followed by an improvement in physical performance.

### Strengths and limitations

This report took advantage of two previously conducted studies of competition in real-world settings where the outcome of the competitions mattered to the participants. Neither study was planned for purposes of studying the coupling of cortisol, testosterone, and estradiol. Each of the studies made a point of determining whether or not participants were using some form of hormone contraception (a strength); oral contraceptive use did not moderate coupling effects with the exception of the soccer players, where estradiol/testosterone was a bit weaker for oral contraceptive users than non-users. Neither study gathered data about the menstrual cycle phases of the participations because the relatively small sample size would have precluded meaningful analysis of cycle phase as related to hormone change during competition. Whether or not menstrual cycle phase affects hormone coupling in women remains an open question to be answered by a larger study intended for this purpose. No attempt was made to determine the hydrational status of the participants or their subjective perception of exertion (physical or psychological)—factors that could conceivably influence levels of the hormones measured for the studies (e.g., Castro-Sepulveda et al., 2018; Maya et al., 2016). Hormone assays for the volleyball and soccer teams were conducted using kits from different suppliers –a fact that presumably contributes to the differences between the two teams with respect to salivary hormone levels (Fig. 2). It is relatively rare that studies of athletic competition include values for estradiol. That the two studies used for this report included values for cortisol, testosterone, and estradiol made it possible to study the extent to which competition-related fluctuations in estradiol are coupled with fluctuations in cortisol and testosterone. The inclusion of assays for estradiol are clearly warranted in future studies of hormone coupling in athletic and non-athletic settings.

### The endocrine correlates of stress: theoretical considerations

The body adaptively responds to stress by the activation of two systems—the autonomic nervous system and the hypothalamic-pituitary-adrenal (HPA axis) resulting in, at least in some circumstances (Turan et al., 2015; Casto & Edwards, 2016a), coordinated elevations in cortisol and testosterone. Women’s athletic competition is associated with an increase in estradiol that appears coordinated with, but not in perfect parallel to, increases in cortisol and testosterone. Going forward, there is an obvious call to more precisely describe the coordinated increases in these three hormones, the variety of conditions under which they occur and the benefits accruing thereof.

## Conclusions

The present study provides evidence that in the hormonally dynamic setting of athletic competition, salivary levels of testosterone and estradiol fluctuate in women athletes over the course of an athletic contest and do so in apparent correspondence with each other. At least for some individuals, estradiol and cortisol also appear to be coupled. Based on an admittedly small sample of two sports—volleyball and soccer—these hormone couplings are probably not sport-specific and would likely be seen in other sports. The extent to which the salivary estradiol couplings reported here occur in non-sport settings and in men as well as women remains to be determined. These results should encourage the sampling of estradiol in addition to testosterone and cortisol in behavioral research and, particularly in studies of status and stress, an exploration of the possible psychological benefits that may accrue from coordinated fluctuations in levels of these hormones.

##  Supplemental Information

10.7717/peerj.8402/supp-1 Supplemental Information 1 Hormone values: 2008 Emory women’s volleyball team For each participant, data points show salivarylevels of testosterone (Test), cortisol (Cort),and estradiol (Est) for samples obtained before and after each of twopractice sessions (P1A, P1B and P2A, P2B, respectively) and before warm-up, at mid-warm-up, and immediately after each of two matches (1A, 1B, 1C and 2A, 2B, 2C respectively). Cortisol is expressed as ug/dl; testosterone and estradiol are expressed as pg/ml. Participants were queried about their use of oral contraception (OC) and identified as either non-users (0) or users (1). For each match, articipants either played (1) or did not play (0).

10.7717/peerj.8402/supp-2 Supplemental Information 2 Hormone values: 2013 Emory women’s soccer team For each participant, data points show salivary levels of testosterone (Test), cortisol (Cort),and estradiol (Est) for samples obtained at neutral-day (Baseline) and before warm-up, after warm-up, immediately after, and 30 min after each of two matches (1A, 1B, 1C, 1D and 2A, 2B, 2C, 2D respectively). Cortisol is expressed as ug/dl; testosterone and estradiol are expressed as pg/ml. Participants were queried about their use of oral contraception and identified as either non-users (0) or users (1). For each match,participants either played (1) or did not play (0).

## Additional Information and Declarations

### Data Availability

The following information was supplied regarding data availability: Raw salivary hormone values are available in the Supplementary Files. Cortisol is expressed as µg/dl and testosterone and estradiol are expressed as pg/ml.
